# Impact of Fabric Construction on Adsorption and Spreading of Liquid Contaminations

**DOI:** 10.3390/ma15061998

**Published:** 2022-03-08

**Authors:** Snježana Brnada, Tanja Pušić, Tihana Dekanić, Stana Kovačević

**Affiliations:** Faculty of Textile Technology, University of Zagreb, Prilaz baruna Filipovića 28a, 10000 Zagreb, Croatia; snjezana.brnada@ttf.unizg.hr (S.B.); tihana.dekanic@ttf.unizg.hr (T.D.); stana.kovacevic@ttf.unizg.hr (S.K.)

**Keywords:** cotton, weave, structure, contamination, dyes, pumpkin oil

## Abstract

A contamination on a textile material is defined as an undesirable, local formation that deviates in appearance from the rest of the material. In this paper the relationship between the shape and surface of liquid contaminations and the firmness factor of woven fabric is investigated. The interdependence of constructional and structural parameters of raw and bleached cotton fabrics were analysed. The results show that selected contaminations are distributed differently, primarily depending on the construction characteristics of the fabric, type of contamination and hydrophilicity of cotton fabric.

## 1. Introduction

Contaminations are various pigments, oils, fats, or dye contaminations, which are undesirable on the textile material and require proper removal [1,2,3]. From a detergency standpoint, contaminations can be categorized as water-soluble substances (e.g., inorganic salts, sugar, urine, and sweat); pigments (e.g., metal oxides, carbonates, silicates, hummus, and soot); oils and fats (e.g., fats of animal origin, fats and oils of vegetable origin, sebum, mineral oils, fats and waxes); proteins (e.g., blood, milk, eggs, and keratin from the skin); carbohydrates (starch); bleachable dyes (e.g., from fruits and vegetables, wine, coffee, and tea). In practice, contaminations mainly occur as combinations of the various types mentioned above [4,5,6].

Various authors have dealt with the problem of textile contamination related to the topographic properties of woven fabric surfaces. The structure of textile materials is very complex and inhomogeneous, which is transferred to their surface properties. In case of woven fabrics, the complexity of the structure arises from the production process that takes place in dozens of production stages—from fibre, through yarn, to flat textile product. For this reason, the surface roughness of the textile material needs to be observed on two levels, distinguishing two types of topography: macro-topography, which refers to the weave (yarn path) and micro-topography which refers to the yarn/fibre surface. For separation of topographic parameters caused by weave and the ones caused by yarn characteristics, a special issue is determining the shortest distance that provides uniform results, also called cut-off length. Various methods for measuring the topographic structure of woven fabrics and the optimization in analysing their complex topographic structure by determining the optimal cut-off length for the separation of two types of fabric roughness, have been proposed by authors [7]. Many scientific studies have been devoted to researching the influence of textile surface roughness on wettability and cleanability, trying to find correlations between topographic parameters and the phenomena of droplet spreading through the fabric surface, wetting, capillary penetration of drops through the fabric, and contamination removal [7,8,9,10]. The findings of all these studies show that the topographic properties of woven fabrics largely depend on their structural and construction parameters. Moreover, a textile surface will have better wettability when it has a certain roughness value, because the capillarity increases with increasing roughness, and the warp micro-roughness plays an important role in fabric cleanability.

In research on the speed of liquid droplets through the textile surfaces, the findings were that the rate of water transfer through the material greatly depends on the structural properties of the fabric, indicating that in case of fabrics with more floating the water transfer through the material is higher [11]. Surface properties of textile material affect the shape and speed of spread of the liquid droplet [12].

Researchers from a wide range of scientific fields take interest in the influence of surface topography on wetting emphasizing that the spectrum of wetting morphologies increases with the complexity of the structures [13,14].

There is an interesting study in the field of forensics [15], which emphasizes the importance of understanding bloodstains on a fabric, since they can provide key information for the interpretation of crimes and accidents: “The formation of bloodstains on non-absorbent surfaces such as glass, as well as on semi-absorbent hard surfaces such as paper, have been studied in depth. However, there has been less research conducted on absorbent surfaces, such as fabrics”. In their study, Kubiak et al. listed factors that influence the shape of a bloodstain on the fabric, such as weave, fibre composition, yarn and fabric structures, surface characteristics, absorptivity, and the manner and amount of maintenance and processing. At the end of their conclusion, they emphasize the need to expand such studies to include different combinations of the above parameters, which would contribute to improved interpretations in forensic research.

When textiles come into contact with a source of contamination, soils are transferred from the source to the textile material. The interaction between the contamination and textile depends on the properties of both the textile material and the contamination.

The surface of the fabrics can be observed at several levels, so adherence of contaminations should also be observed at several levels: micro, mini, meso, and macro.

At the micro-level, the mechanical of adhesion of contamination onto textile material depends on the strength of interaction forces between the fibre and the contamination, where the polarity of fibres also affects the adhesion potential. In aqueous solutions, the fibre is characterized by zeta potential, an important parameter for the propensity to contaminate [1,16].

**Figure 1:**
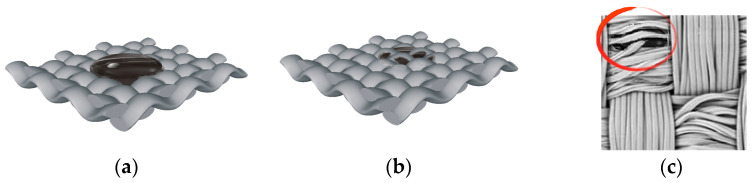
Levels of interaction between contamination and woven fabric. (**a**) Surface adhesion of contamination on the fabric; (**b**) liquid contamination penetration into the fabric structure; (**c**) liquid contamination penetration into the structure of the yarn.

The meso level implies a material, e.g., fabric structure, with a more or less porous surface and mostly irregular interspaces in the fabric. Figure 1a shows a simulation of fabric and contamination made in TexGen software. The interaction between the textile fabric and the contamination is related to the coefficient of adhesion between the contact area of the fabric and the contamination.

Meso level implies the penetration of contamination within the structure of woven fabric and yarn. The compactness of the structure of plied yarns is relatively lower than that of single spun yarns. Therefore, the generated cavities (channels, gaps) facilitate the penetration of liquid contamination into the structure of the plied yarn. In the case of fabrics, the crossing points of the warp and weft yarns can be singled out as potential barriers to the penetration of contamination through the fabric structure, where the planarity of the surface and mechanical retention of contaminations are crucial, Figure 1b.

The mini level refers to the fibrillar yarn structure of textile materials. Figure 1c shows yarn structure, weave, the yarn type (single), and four crossing points with the highlighted segment of the contamination deeply penetrating the fibres.

In the case of penetration of liquid contamination into the structure of the yarn, it is important to consider the contamination particle size, e.g., the pigment, which must be smaller than the interspace between the fibres in the yarn. Liquid contaminations must have a low surface tension to penetrate the interspaces, as well as low viscosity to spread and distribute, Figure 1c.

Mechanical removal of contaminations can cause discoloration at the micro-level (fibre). In this case, the contamination penetrates into the structure of the fabric, yarns, and finally the fibres where it enters into a chemical reaction and ties in by certain forces. This type of contamination can be removed only through chemical or enzyme mediation of agents.

**Figure 2:**
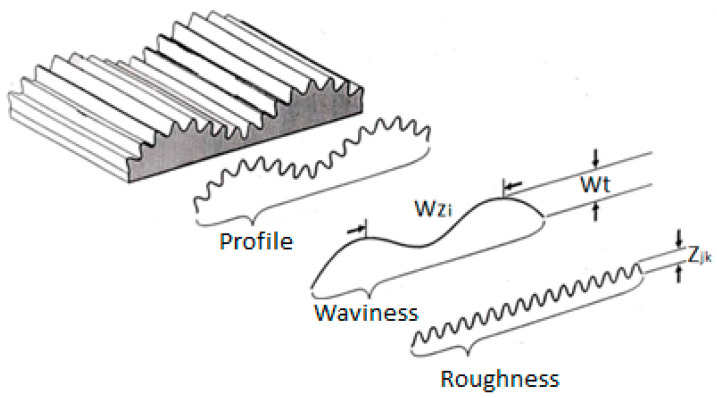
Display of waviness and surface roughness of the material [18].

Topographic properties of surfaces are characterized by surface roughness and waviness parameters [7,17,18]. In fabrics, the typical topography is expressed by the regular repetition of waves in the vertical, horizontal or off-axis directions which result from a specific structure. The structure of the fabric is defined by the construction parameters of the yarn, the weave (type of weave and the size of the weave unit) and the yarn density in the warp and weft directions. However, to establish the relationship between fabric design parameters and the tendency towards contamination, it is necessary to analyse the fabric’s surface properties at the meso level. The topographic properties of woven fabrics at this level are the result of the weave, structural properties, and the type of yarn. The parameters that describe the topographic characteristics of the material are reflected in the characteristics of the waviness and roughness, Figure 2 [18].

Roughness is defined as closely spaced irregularities, while waviness refers to widely spaced irregularities on the surface of the material.

Where Wt is the wave height, defined as the distance between the highest and lowest point of the wave W; Wz is the wavelength [18].

**Figure 3:**
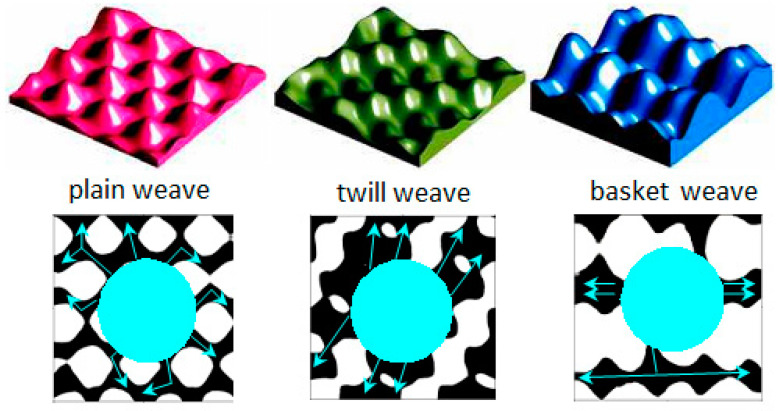
Topographic structure of fabrics in plain, twill and basket weave and the direction of movement of liquid droplets on the fabric surface [17].

In the research of Calvimontes et al. [17], the correlation between the topographic parameters of woven fabrics and the velocity and direction of liquid droplets’ movement on the material was investigated. Topographical differences were found between samples woven into plain, twill, and basket weaves. In woven fabrics with the highest values of the waviness, the spread of liquid droplets was the slowest. In twill weave fabrics structured as so-called “channels” with “islands”, the droplet speed was higher than in the plain weave sample. In the basket weave samples, the topographic structure has continuous “channels” (without “islands”) and the rate of droplet distribution was the highest. Illustrations of these weaves are presented in Figure 3. Analogously, liquid contaminations will be distributed more rapidly across fabrics with higher weave factor values (loose weaves).

Structural factors of fabrics are always calculated as the ratio of a certain mathematical equation of the parameters of the observed fabric to the same parameters for the standard fabric in a plain weave. Milašius et al. [19] proposed a new integrating fabric firmness factor that can be calculated by Equations (1)–(3),

(1) $$P_{1(2)}=\sqrt{\frac{3\cdot R_{1}\cdot R_{2}}{3\cdot R_{1}\cdot R_{2}-\left(2\cdot n_{f1(2)}+\sum_{i=1}^{6}K_{1(2)i\cdot }n_{f1(2)i}\right)}}$$

where *P*_1_ is the weave factor, *R*_1_ and *R*_2_ are repetitions of the warp and weft in the weave unit, *n_f_* is the number of free fields (flotation), *n_fi_* is the number of free fields belonging to group *i*, and *K_i_* is the elimination factor of group *i*.

Milašius equation:

(2) $$\varphi =\sqrt{\frac{12}{\pi }}\cdot \frac{1}{P_{1}}\cdot \sqrt{\frac{T_{average}}{\rho }}\cdot {S_{2}}^{\frac{1}{1+2/3\sqrt{T_{1}/T_{2}}}}\cdot {S_{1}}^{\frac{2/3\sqrt{T_{1}/T_{2}}}{1+2/3\sqrt{T_{1}/T_{2}}}}$$

(3) $$T_{average}=\frac{T_{1}\cdot S_{1}+T_{2}\cdot S_{2}}{S_{1}+S_{2}}$$

where *T*_1_, *T*_2_, and *T_average_* are the warp and weft fineness, and average thread fineness (tex); *P*_1_ is the Milašius weave factor; *ρ* is the fibre density; and *S*_1_ and *S*_2_ are warp and weft density at 10 cm.

Testing of contamination resistance is performed through intentional deposition of contamination on the textile material. Because there are many different types of contamination, there is no standardized method for testing the tendency of textiles towards contamination. However, different institutions use internal methods to test materials under specific conditions [18].

Spectral evaluation determines the effect of contamination removal (S) or discoloration (degree of lightening) after a certain number of wash or dry-cleaning cycles [1].

According to [17], the rate of surface distribution and penetration of oil contaminant ions into textiles can be tested by (a) measuring the surface distribution of the inert oil drop into a textile by microscope [20] and (b) examination of the surface distribution of oil droplets (inert paraffin) on the textile material by observing the shape of a drop from the side and estimating its height and appearance at certain intervals (oil repellency assessment).

The wetting of textile material takes place in several stages [21,22,23]: (a) dynamic wetting, manifested by an increase in the ground plan diameter of the oil/water droplet on the textile material per unit time (spread of the droplet); (b) quasi-static wetting, in which the dimensions of the droplet are constant in a unit of time; and (c) penetration of the droplet into the fabric structure (water repellence assessment).

Numerous colourants and fluorescent compounds as contamination substances can be used for lignocellulosic fibres, as well as their chemical components, cellulose, hemicellulose and lignin. Methylene blue and Benzopurpurine 4B, among other colourants, are specific to cellulose fibres [24]. Reference soils (blood, cocoa, pigment, fat, starch, etc.) always appear on standard fabrics weaved in the canvas, which does not provide sufficient insight into the behaviour and properties of reference soils on other fabric weaves, although it is essential for a more comprehensive assessment of the adherence and removal of specific stains [25].

According to the literature, it can be concluded that the weave type, i.e., the proportion of air space between the thread and the fibres, affects the intensity of liquid spread.

In this paper a wider range of fabric constructions, in different warp and weft densities and different hydrophilicity, treated by contaminations of different types are investigated. The rate of spread and penetration of a liquid contamination at the place of dripping, during and after the spreading, is analysed. Moreover, the way in which liquid contamination penetrated the fabric structure was investigated. Shape deviations away from round depend primarily on the structural parameters of the fabric, such as weave, density, and fineness of the threads, and the material composition.

The relationship between the contamination shape and the surface firmness factor of the woven fabric, which is directly related to the number of interlacing points of the warp and weft threads, is investigated in this paper for the first time.

## 2. Experimental Part

### 2.1. Materials and Specimens for Testing

Woven fabric samples, produced on the same weaving machine (Picanol OMNIplus 800, air-jet weaving machine, width 190 cm in the textile factory Čateks d.o.o., Čakovec, Croatia) and from the same warp production series, were tested. The same yarn was used for the warp and weft: composition 100% cotton, combed, single yarn, fineness 35.7 tex, 505 S twist/m, produced in the same production batch. The fabric was woven in seven different weaves and two different warp and weft densities, specified in Table 1. The numerical part of the samples’ designations refers to the density of the warp and weft, i.e., 44 stands for the samples with a warp/weft density of 24/24 threads per centimetre, while 00 stands for the samples with a warp/weft density 20.2/20.2 threads per centimetre. As can be seen from the data, the bleaching process had a negligible effect on warp and weft density. Half of the tested samples were bleached in a stabilised bath of hydrogen peroxide with the addition of alkali and a wetting agent. The bleaching recipe was: 4 mL/L H_2_O_2_ (hydrogen peroxide); 2 mL/L NaOH (30%); stabilizer and wetting agent, 2 g/L Viscavin CCP (CHT). Treatment of the material was performed in a bath ratio of 1:10, at 98 °C, for 45 min in a laboratory device (Turbomat P4502, Mathis, Oberhasli, Switzerland). After the treatment, the samples were washed with hot water (85 °C) followed by two cycles of cold water, neutralized with acetic acid (1% acetic acid) and washed until neutral. Drying was carried out in the free state at 105 °C, by air flow in a drying chamber (Nr. 6235, Scholl AG, Zofingen, Switzerland). The other half of fabric samples remained raw.

**Figure 4:**
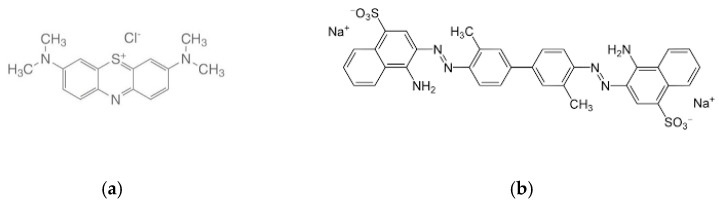
Chemical structure of (**a**) Methylene blue and (**b**) Benzopurpurine 4B.

Samples were contaminated with three liquids: water solution of dyestuffs, prepared by addition of 0.1 g of dyestuff in a solution of non-ionic surfactant (0.1 g/L) and pumpkin oil. Blue dye solution—Methylene blue (C.I. Solvent Blue 8, C.I. 52015), molecular structure: thiazine class, Figure 4a. Red dye solution—Benzopurpurine 4B (C.I. Direct Red 2, C.I. 23500), molecular structure: double azo class, Figure 4b.

The dyes selected as contaminants are referred to as red dye and blue dye in the Results and Discussion sections.

### 2.2. Methods

#### Measurement of Droplet Penetration

In order to determine the rate and direction of one drop of liquid contamination distribution, the samples were recorded using a camera. Two different fabrics: raw and bleached, and three types of liquid contamination: blue dye, red dye, and pumpkin oil, were used.

The advantage of these two colouring agents is that their chemical structure is known and that they have high purity and an affinity towards cellulose substrates. Adsorption of Methylene blue in liquid form has been studied in a study of the surface area of 6 reference cotton fibres. The results of this study confirmed the relevance of this method for the characterization of cotton fibres in quality control, similar to other mechanical methods.

In addition, the adsorption amount of the cationic dye Methylene blue and anionic dye Benzopurpurine 4B to cotton can be used to assess and differentiate the adsorbate properties regarding the influence of structural parameters and the degree of finishing, which is important for this study. The study is designed to determine the behaviour of dye solutions of different structure, planarity, and ionogenicity, with Methylene blue as a cationic and Benzopurpurine 4B as an anionic dye.

The fabric sample was placed on a flat transparent smooth surface with a video camera underneath the table.

One drop of liquid contamination of known volume was applied to the sample with a burette from a height of 30 mm. The behaviour of the drops was monitored with the camera (digital microscope DinoLite, Premier IDCP B.V., Almere, The Netherlands), from the bottom of the transparent surface. After the subjective assessment of completion of the droplet’s propagation, video recording was stopped.

The videos were divided into photos, with 15 photos per second of video. The starting point was determined with an accuracy of 0/15 = 0.067 s, i.e., from the moment of initial contact of the droplet with the sample. An analysis of the first 5 s of droplet distribution per sample at 20 s spacing was performed. All the images from the video (15 fps) were isolated and every third photo was taken to show the appearance of the contamination every 20 s. The first 5 s (every 20th hundredth) were selected from the set of photos, and every 50th second after that until the end.

Image processing was performed using the Fiji software, released as open source under the GNU General Public License, allows converting an image to a black and white projection using various filters, making it easier to analyse the contaminated surface [26]. The selected procedure was as follows: a set of photos from one video was combined into one stack which was treated equally. The resulting set of black-and-white images was passed through a filter that showed all the pixels darker than the default hue in black and lighter in white. Binary projection of the contamination image, with prior calibration in which the connection of the number of pixels with the real unit area on the image is established, allows to measure the change in the area of contamination over time. Droplet spreading propagation is expressed as a percentage by calculating the increase in the number of black pixels at moment “n” compared to moment “0”.

## 3. Results and discussion

### 3.1. Weave and Firmness Factor

Weave and firmness factor for raw and bleached fabrics in weave P1/1, Pa2/2, K1/3, K2/2, A1/4 (3), R1/1 (2 + 2), and R2/2 (1 + 1) in two densities are shown in Table 2.

The bleached fabric in plain weave with warp and weft density of 24 threads/cm had the highest values of firmness factor, while the raw satin fabric had the lowest compactness in with warp and weft density of 20 threads/cm. The rate of contamination spreading through the material as well as the direction of distribution depended on the weave factor. Warp and weft rib weaves belong to the group of so-called unbalanced weaves, which is characterized by unequal interlacements of warp and weft threads in the weave unit. Therefore, the weave and firmness factor in warp and weft directions were calculated separately.

**Figure 5:**
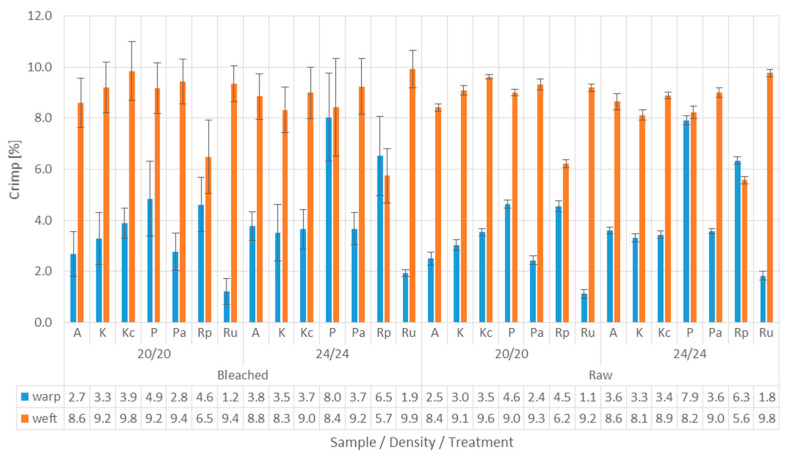
Warp and weft crimp in fabric samples.

Crimp and the relationships between the weave, density and processing of fabrics are shown on Figure 5.

The graph in Figure 5 shows that the warp crimp values are lower than the weft crimp values, which is in line with fundamental textile theory. Due to the shrinkage of the fabrics in the bleaching process, the crimp had changed slightly, which affected the topographic structure of the woven samples’ surface and consequently the behaviour of contamination drops.

### 3.2. Determination of the Droplet Penetration Rate

**Figure 6:**
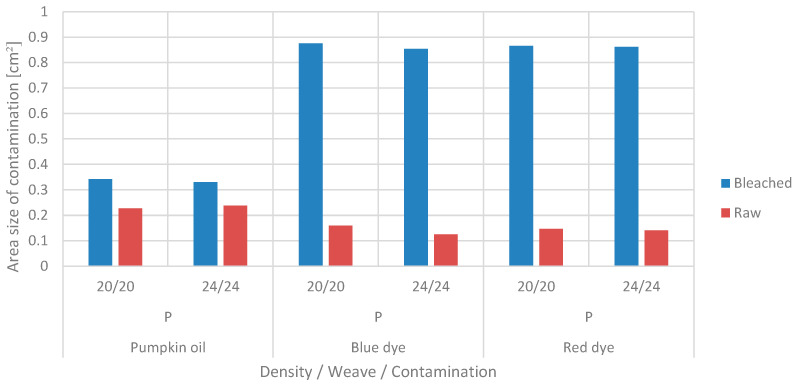
The area of drops (in cm^2^) on bleached and raw samples in plain weave, with densities of 20/20 threads/cm and 24/24 threads/cm.

Surface affinity of woven fabric to contamination, varies depending on structural and constructional characteristics. A particular contaminated area of fabrics in a plain weave was determined after the droplet stopped spreading. Plain weave is a basic weave selected as a standard, with a maximum number of interlacements, minimum weave unit area and highest strength and stiffness. The area of the drops on the raw and bleached fabric samples in plain weave fabrics, densities 20/20 and 24/24, is presented in Figure 6. 

The surface area values of the red and blue dyes on the bleached samples were the highest. The contamination surface area of these dye solutions on the raw sample was significantly lower. The surface area of pumpkin oil was also larger in the case of the bleached sample. In addition, the differences in the areas of contamination with pumpkin oil in the raw and bleached samples were significantly smaller than in the dyed ones.

The raw cotton fabric was hydrophobic due to wax and other impurities, so the spreading and penetration of lyophilic liquid, i.e., pumpkin oil, was greater than in bleached fabric. In the process of scouring and bleaching the cotton fabric, hydrophobic substrates and pigments were removed, thereby increasing its hydrophilicity, i.e., its affinity for the applied dye solutions.

**Figure 7:**
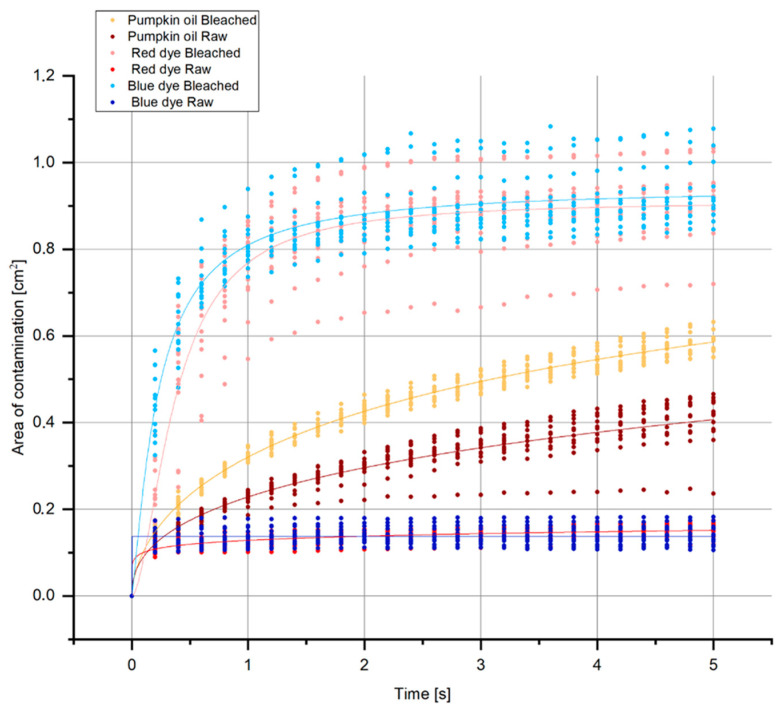
Change in contamination area size (average area size of contamination in cm^2^) regardless of weave and fabric density in the first 5 s.

Figure 7 shows the distribution of the droplets in the first 5 s grouped by type of contamination and type of treatment, regardless of weave and density. Penetration and distribution of red and blue droplets were faster on the bleached samples than on the raw ones. The equilibrium state of the dye solution (stop in spreading) was achieved in all samples. Therefore, pumpkin oil was not balanced on all surfaces after the first 5 s. This may be explained by oil viscosity and hydrophobicity, which certainly slows down the spread.

**Figure 8:**
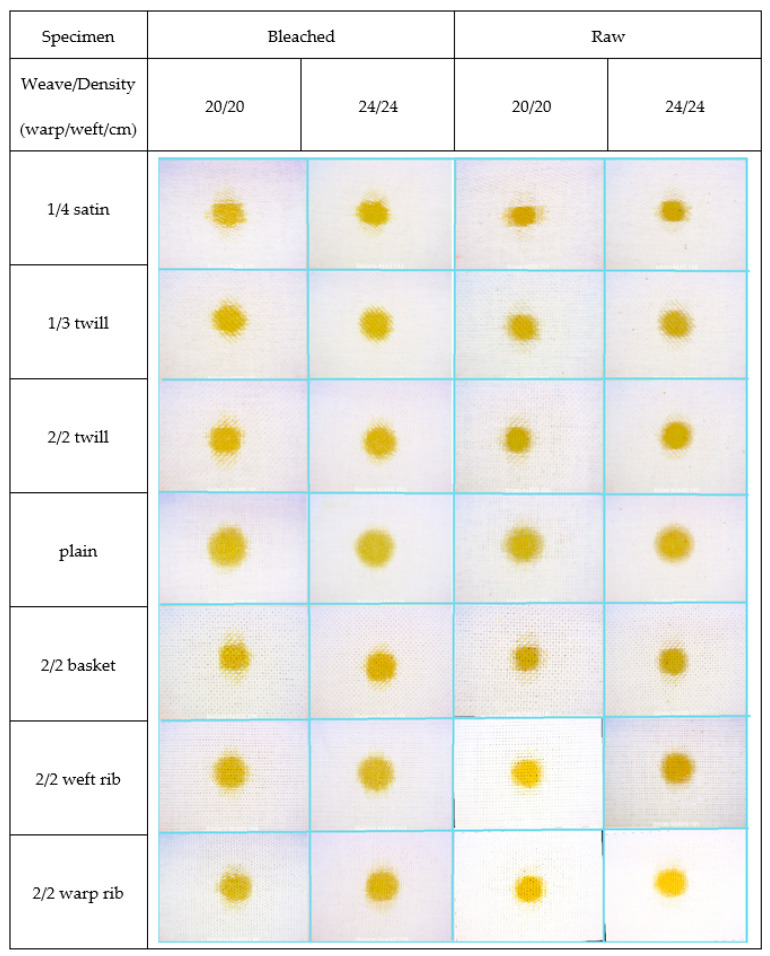
Images of raw and bleached specimens at the end of spreading of pumpkin oil droplets.

**Figure 9:**
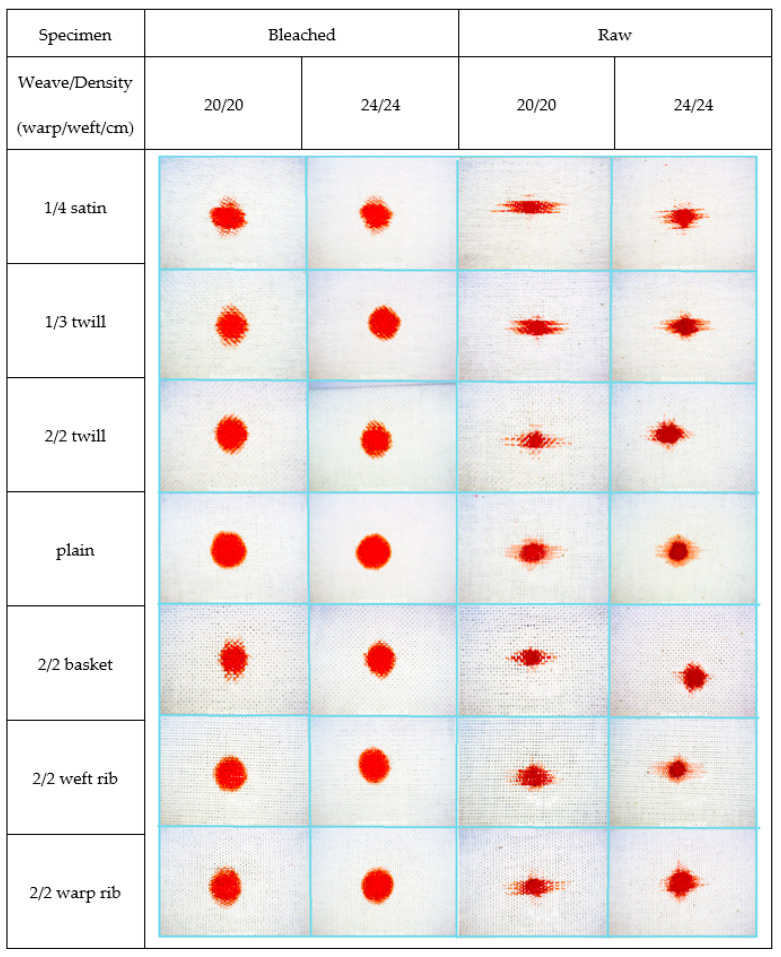
Images of raw and bleached specimens at the end of spreading of red dye droplets.

**Figure 10:**
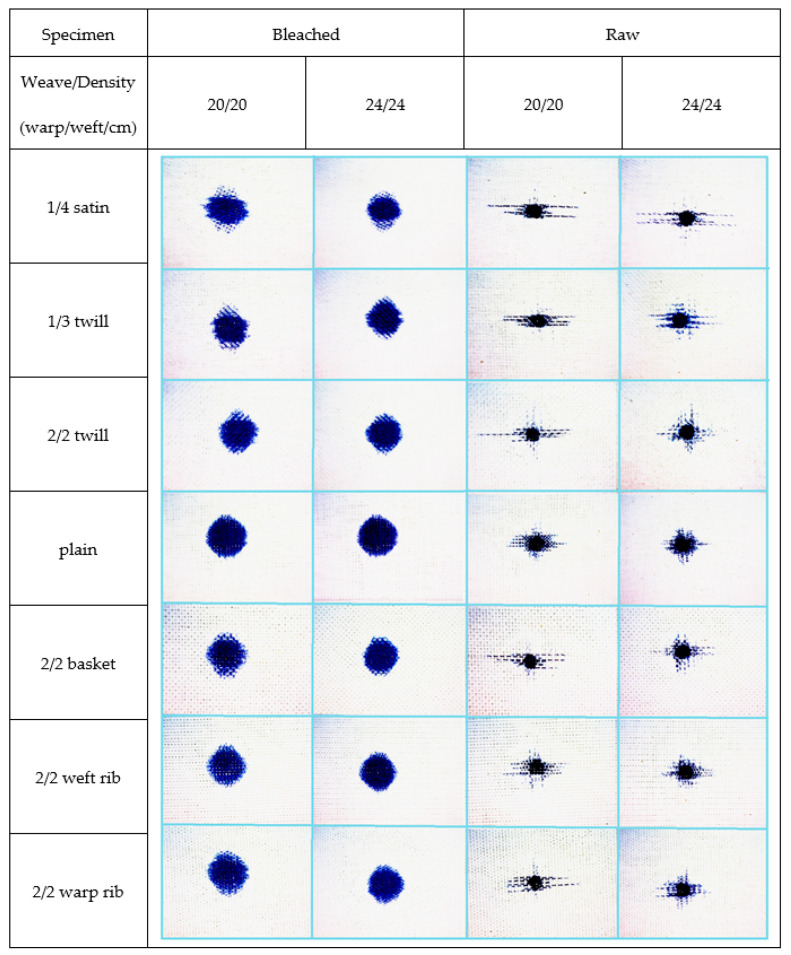
Images of raw and bleached specimens at the end of spreading of blue dye droplet.

Figure 8, Figure 9 and Figure 10 show the bleached and raw fabric specimens with a drop of contamination, observed at the moment the spread stopped, i.e., when the increase in the number of pixels in a period of 1 s did not exceed 5%. All images were taken at 62× magnification.

The following levels of droplet distribution were identified on the rough surface (fabric), consisting of linearly directed yarn threads and composed of a bundle of fibres of a specific length twisted torsionally:

K—distribution of drops on the rough surface of the fabric through “channels” created as a result of the weave;

O and P—penetration of drops into the yarn structure and capillary penetration between fibres in the yarn in the direction of the warp (O) and weft (P);

V—penetration of droplets into the fibre and distribution by transfer to nearby fibres.

The distribution of drops was categorized into three levels of distribution and depends on the affinity of the drops for the fibre surface. Table 3 shows the penetration of contamination into the fabric specimens depending on the parameters of the fabric structure, treatment, and type of contamination.

The distribution along the channels extends in the direction of diagonal stripes at an angle of 45° to the warp direction.

Capillary penetration through the yarn was characterized by the absence of contamination distribution in directions other than the warp and weft directions. Capillary penetration can be more prominent in the warp or weft direction, which are analysed separately. This type of contamination distribution has a characteristic streaked appearance on the edges, and the contamination has an orthotropic appearance.

The absorption of liquid droplets into the fibre and its expansion was manifested in a uniform, circular form of contamination after cessation of expansion. The contamination expanded isotropically and was not affected by the constructional characteristics of the fabric.

Fabric samples in the satin weave “absorbed” all types of contaminations exclusively by capillary action. In case of raw fabrics samples, the effect was more pronounced in the weft direction, while in bleached samples the absorbance was more pronounced in the warp direction. It should be emphasized that the woven fabric’s front side was dominated by the weft effect in a ratio of 4:1. Moreover, the surface of the red and blue dye contamination on bleached samples (circle) had a much larger radius compared to raw samples, which means that the contamination was first distributed by absorption into the fibre to a certain distance from the centre, after which it continued to move through the yarn by capillary action.

In the case of fabric samples in twill weaves, K1/3 and K2/2, which are diagonally structured, it was expected that their properties would not take extremes in the warp and weft directions. Therefore, a contamination distribution in the diagonal channels took place in bleached specimens treated with blue and red dye and pumpkin oil. In the case of raw specimens, this distribution was evident only in the case of contamination with pumpkin oil, while in the case of contamination with red and blue dye, in both twills, the distribution was capillary through the yarn with a more noticeable effect on the weft direction.

The fabrics in plain and basket weave are symmetrical in their structure, and have an equal share of warp and weft crossing points with a relatively homogeneous rough appearance and small repeating elements. Fabrics in plain weave had the maximum number of interlacements and so represent the reference for comparing the properties of fabrics in other weaves. Differences in properties in the warp and weft direction of selected fabrics in plain and basket weave could be solely the result of different warp and weft crimp and a possible deterioration of mechanical characteristics of the warp due to cyclic and tensile stresses in the weaving process. Fabrics in plain weave displayed high values of compactness, but low values of the cover factor. Bleached fabric specimens absorbed all applied contaminations by absorbing them into the fibres and transferring them to the adjacent fibres.

In the case of raw specimens, the contamination with pumpkin oil behaved in the same way, while in the case of dyes there was a combination of absorption into the fibre and continuation by capillary transfer through the yarn, with an enhanced effect mainly in the weft direction.

Fabric specimens in basket weave were identical in their composition to plain weave, but with a higher coverage factor and less compactness. The fabric in basket weave had twice fewer interlacements than the fabric in plain weave, whereas the yarn was less tight in the fabric. Due to the lower structure compactness of specimens in basket weave in comparison to plain weave, there was no transfer of contaminants through the fibres. The contamination droplet spread exclusively by capillary action, through the yarn.

For bleached samples of all densities and contamination types, the distribution was pronounced in the direction of the warp. On the other hand, for higher densities (24/24) in the case of dye contaminations, there was also distribution in the weft direction. In the case of raw specimens in basket weave, pumpkin oil was distributed exclusively in the warp direction. Contamination of raw specimens in the basket weave depended on density: at lower density (20/20) the capillary distribution was pronounced in the weft direction, while at higher density (24/24) it was equally pronounced in both the warp and weft directions. 

The fabrics in the rib weave are different compared to the other analysed weaves in the manner of interlacements in warp and weft direction.

In the case of raw samples in the warp and weft of rib weave specimens, the application of drops of pumpkin oil resulted in capillary penetration with a pronounced effect in the warp direction, at both densities. The distribution of red and blue dye to specimens of warp and weft ribs at lower density was also by capillary action through the yarn, but was more pronounced in the weft direction, while at higher fabric densities, the distribution was by capillary action and even in the warp and weft directions.

When applying blue dye to raw fabrics, in all samples except the satin, the distribution of contamination was capillary through the yarn. In 20/20 density samples the distribution was more pronounced in the weft direction, while in 24/24 density samples the distribution was equal in both directions.

### 3.3. Representative Examples of the Drop Distribution on the Rough Surface of the Fabric

#### 3.3.1. Distribution of Drops on the Relief Surface of the Fabric along the “Channels” of Diagonally Structured Fabrics Created as a Result of the Weave Structure

**Figure 11:**
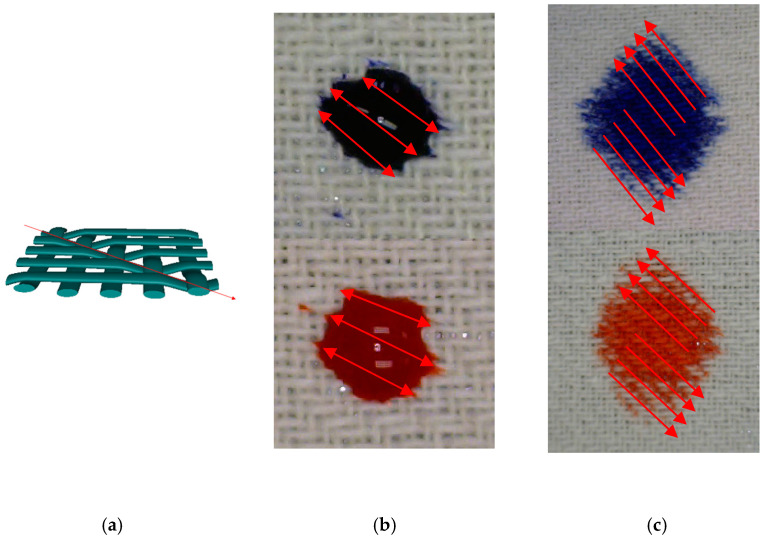
Diagonal “channels” on the surface of the fabric in twill 1/3 weave: (**a**) twill weave 3D simulation. Distribution of blue and red dye drops through the diagonals of the fabric in the twill weave: (**b**) start position, (**c**) end position.

Figure 11a shows a simulation of fabric in twill weave, where the diagonality in the structure in relation to the main axes of the fabric (direction of the warp and weft) is clearly visible.

On a relief surface of this type of fabric, a diagonal structure appears, where the pronounced diagonals are channels that have their lowest point in the middle axis, i.e., it represents the lower part of the wave. Consequently, the path of movement of the liquid through the hydrophobic surface of the fabric in twill weave was exactly in the direction of these diagonals. An example of this is shown in Figure 11b,c. 

Figure 11b,c shows the diagonal migration of dye droplets on a bleached twill 1/3 fabric, density 24/24. The left figure b shows a drop at the very beginning of the distribution, where it can be seen that the drop took an elliptical shape, rotated by 45°. The right figure c shows the sample after the contamination spreading ended.

#### 3.3.2. Penetration of Drops into the Yarn Structure and Capillary Penetration between Fibres in the Yarn

**Figure 12:**
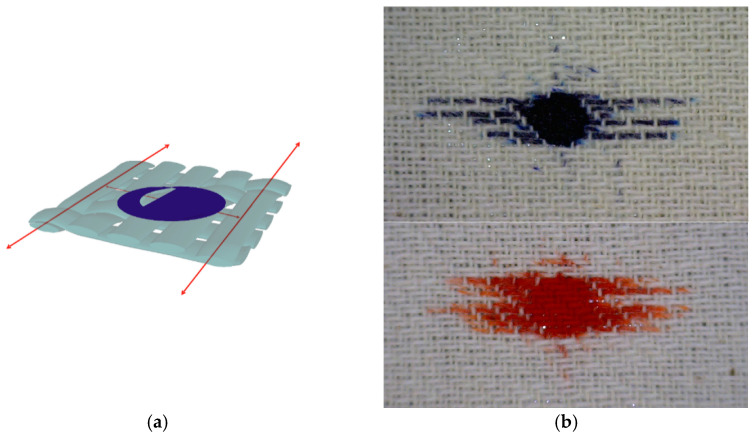
Level 2-drop distribution in yarn, (**a**) 3D simulation, (**b**) penetration of blue and red dye droplets into the yarn structure and capillary distribution through yarn.

The penetration of the droplets into the yarn structure and the capillary penetration between the fibres in the yarn are shown in simulation on Figure 12 on the example of a hydrophilic fabric in a twill weave.

Figure 12b shows a sample of raw fabric in twill 1/3, density 20/20. It can be seen that the dye, after penetration into the yarn structure, was transported by capillary action across the yarn. The transfer of dye between fibres is negligible—the warp threads on the cross points were completely uncoloured because of their higher stiffness, even where the weft that passes under and touches them completely absorbed the dye. The effect was pronounced in the weft direction. The reason for this may be the greater compactness of the warp threads compared to the weft, due to high stresses in the weaving process, as the warp, unlike the weft, is very tense in the weaving process. The higher crimp of the weft was the result of high warp tension in the weaving process. As a result, the weft adapts more to the warp, while the warp remains a tighter system in the finished fabric; the fibres inside are tighter, which leaves little a space for the dye.

#### 3.3.3. Penetration of Droplets into the Fibre and Distribution by Transfer to the Fibres in Contact

On hydrophilic fabric, the absorption of liquid contamination into the fibre took place at such a rate that other types of droplet migration along the relief surface of the fabric or capillary penetration into the yarn structure could not occur. The importance of weave and structure in this case was almost insignificant because the distribution of liquid drop on the hydrophilic surface was transmitted by direct contact from fibre to fibre.

**Figure 13:**
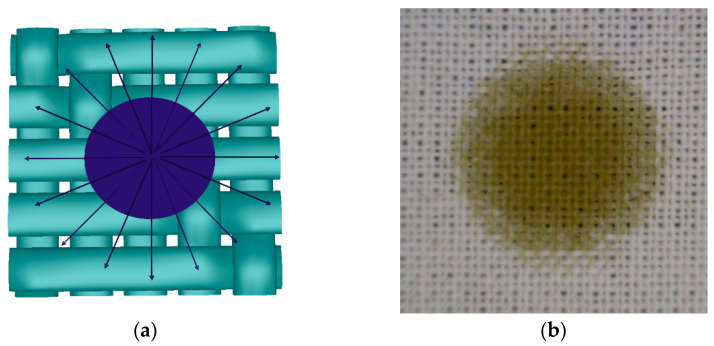
Distribution of drops with a uniform tendency to spread from the centre outwards: (**a**) 3D simulation; (**b**) Penetration of a pumpkin oil drop into the fibre and transfer to adjacent fibres—proper distribution, equal in all directions (bleached fabric in plain weave, density 24/24).

Figure 13a shows a simulation of a fabric with a uniform and symmetrical appearance of contamination distributed on its surface. A uniform circle was the result of good permeability of the liquid contamination through the material by easy transfer from fibre to fibre.

Figure 13b shows a bleached fabric in a 24/24 density contaminated with a droplet of pumpkin oil.

A larger amount of oil is visible in the middle of the contamination in the form of a darker shade, while this amount decreases towards the edges, where the contamination is lighter. This shows the fibre to fibre transfer of the droplet through the material where the oil penetration towards the edges was weaker due to the oil viscosity. This type of distribution was influenced by the compact structure of the fabric in the plain weave with a high firmness factor and a high affinity of bleached fibre to the pumpkin oil.

## 4. Conclusions

The results show that, as far as the affinity of different contaminations for fabrics is concerned, pumpkin oil had a less pronounced affinity for raw samples than bleached fabrics. When it comes to the affinity of the material for dyestuff contaminations, bleached samples had a faster distribution of dye, while raw samples had a slower absorption. The contamination of the surface depends more on the treatment of the fabric and the type of contamination than on the weave.

The contamination distribution on the surface of textile materials was determined by the speed and direction. While the speed of absorption was largely determined by the degree of finishing of the fabric and the type of contamination, the direction of movement of the contamination depended on the hydrophilicity of the fabric and its constructional and structural characteristics. It is important to note that the levels of contamination distribution described in the theoretical part of this article are ideal cases with clear examples. In the experiment, a combination of two or even three levels of distribution occurred in most contaminations. In the case of diagonally structured fabrics of lower hydrophilic character, the distribution of the contamination was intense in the directions of the diagonals, and the final form of contamination had an elliptical, twisted shape. A pronounced capillary distribution through the yarn (threads) of the warp and weft was present in the raw samples contaminated with dyestuff. Therefore, greater dye penetration was taken over by a less tense system with higher crimp values (mostly weft). The distribution of fibre-to-fibre transfer movements with consequent isotropic contamination propagation occurred in cases of strong affinity of the textile material to the type of contamination, i.e., in the following cases: bleached fabrics—dyestuff (red and blue) and raw fabrics—pumpkin oil.

The phenomenon of spreading liquid contamination through the fabric surface was visible in all weave types, raw materials, and types of contaminations, which indicates that weave, density of woven fabric, the raw material composition and hydrophilicity, significantly affect the shape and size of liquid contamination. Regardless of the marked differences in ionogenicity, chemical, and structural characteristics, no significant differences in the behaviour of blue and red dye were found. The frequency of interlacements of the warp and weft threads into the weave (the plain weave has the maximum number of interlacements, and the satin weave has the smallest number of interlacements) also affects the shape and amount of liquid contamination on the fabric. The higher frequency of warp and weft interlacements affects the more difficult penetration of liquid contamination.

## Figures and Tables

**Table 1:** Woven fabric samples labels, weave and thread densities.

Designation	Weave	Declared Density (Threads/cm)	Determined Warp Density g_wa_ (Threads/cm)		Determined Weft Density g_we_ (Threads/cm)	
			Raw	Bleached	Raw	Bleached
P44	Plain	24.0/24.0	23.6	24.0	23.6	24.2
Pa44	2/2 basket		24.0	24.0	23.0	23.2
Kc44	2/2 twill		24.0	24.0	23.0	24.0
K44	1/3 twill		23.6	24.0	23.8	24.2
A44	1/4 satin		23.8	24.0	23.8	24.0
Ru44	2/2 weft rib		24.0	24.5	23.4	24.0
Rp44	2/2 warp rib		23.4	23.6	23.8	24.5
P00	Plain	20.2/20.2	20.2	21.0	20.0	20.0
Pa00	2/2 basket		20.0	20.0	19.8	21.0
Kc00	2/2 twill		20.0	20.0	19.5	20.2
K00	1/3 twill		20.0	21.0	19.5	20.8
A00	1/4 satin		20.0	20.8	19.8	20.5
Ru00	2/2 weft rib		21.0	21.0	19.0	20.0
Rp00	2/2 warp rib		20.0	20.0	20.2	21.0

**Table 2:** Weave and firmness factors.

	Plain Weave	2/2 Basket	2/2 Twill	1/3 Twill	1/4 Satin	2/2 Weft Rib	2/2 Warp Rib
	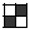	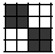	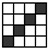	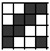	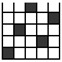	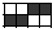	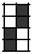
P1(2)	1.000	1.359	1.333	1.265	1.414	1.309/1.000	1.000/1.309
φ, % (20/20) raw	60.9	44.8	45.7	48.2	43.1	46.5/ 60.9	60.9/ 46.5
φ, % (24/24) raw	73.1	53.8	54.8	57.8	51.7	55.8/ 73.1	73.1/ 55.8
φ, % (20/20) bleached	62.5	46.0	46.8	50.6	45.2	47.7/ 62.5	62.5/ 47.7
φ, % (24/24) bleached	74.5	54.9	56.0	60.2	53.9	57.0/ 74.6	74.6/ 57.0

**Table 3:** Penetration of contamination (direction, rate, and level of absorption) depending on the fabric weave, density, and type of contamination.

Density (Warps/cm/Weft/cm)	Contamination	Treatment	Plain Weave	2/2 Basket	2/2 Twill	1/3 Twill	1/4 Satin	2/2 Weft Rib	2/2 Warp Rib
20/20	Pumpkin oil	Bleached	V	O	K	K	O	O	O + P
		Raw	O + V	O	K	K	O + P	O	O
	Red dye	Bleached	V	O	K + V	K	O	O	O + P
		Raw	P	P	P	P	P	P	P
	Blue dye	Bleached	V	O	K + O + P	K	O + P	O + P + V	O + P
		Raw	P	P	P	P	P	P	P
24/24	Pumpkin oil	Bleached	V	O	K	K	O	O	O + P + V
		Raw	V	O	K	K	O + P	O	O
	Red dye	Bleached	V	O	K	K + V	O	V	V
		Raw	V + P	O + P	P	P	P	O + P + V	P
	Blue dye	Bleached	V	O	K + O + P	K	O + P	O + P + V	O + P + V
		Raw	O + P	O + P	O + P	O + P	P	O + P + V	O + P

## Data Availability

The data are open available.

## References

[1] Rouette H.K., Schwager B. (2001). Encyclopedia of Textile Finishing.

[2] Toure Y., Mabon N., Sindic M. (2013). Soil model systems used to assess fouling, soil adherence and surface cleanability in the laboratory. Biotechnol. Agron. Soc. Environ..

[3] Dhiman G., Chakraborty J.N. (2014). Soil release performance of cotton finished with Oleophobol CPR and CMC-Na salt. Fash. Text..

[4] Smulders E., Rahse W., Rybinski W. (2002). Laundry Detergents.

[5] Ishikawa Y., Oya M. (2008). Application of Statistical Analysis to Mixed Soil Detergency. J. Oleo Sci..

[6] Oya M., Taniguchi Y., Fujimura N., Miyamoto K., Oya M. (2020). Kinetic analysis of hemoglobin detergency by probability density functional method. PLoS ONE.

[7] Calvimontes A., Badrul Hasan M.M., Dutschk V. (2010). Effects of topographic structure on wettability of woven fabrics. Woven Fabric Engineering.

[8] Badrul Hasan M.M., Calvimontes A., Dutschk V. (2009). Correlation between wettability and cleanability of polyester fabrics modified by a soil release polymer and their topographic structure. J. Surfactants Deterg..

[9] Calvimontes A., Dutschk V., Stamm M. (2010). Advances in topographic characterization of textile materials. Text. Res. J..

[10] Calvimontes A., Saha R., Dutschka V. (2011). Topographical effects of O_2_-and NH_3_-plasma treatment on woven plain polyester fabric in adjusting hydrophilicity. Autex Res. J..

[11] Lei M., Li Y., Liu Y., Ma Y., Cheng L., Hu Y. (2020). Effect of Weaving Structures on the Water Wicking–Evaporating Behavior of Woven Fabrics. Polymers.

[12] Romdhani Z., Baffoun A., Hamdaoui M., Roudesli S. (2014). Drop impact on textile material: Effect of fabric properties. Autex Res. J..

[13] Seemann R., Brinkmann M., Herminghaus S., Khare K., Law B.M., McBride S., Kostourou K., Gurevich E., Bommer S., Herrmann C. (2011). Wetting morphologies and their transitions in grooved substrates. J. Phys. Condens. Matter.

[14] Kubiak K.J., Wilson M.C.T., Mathia T.G., Carval P. (2011). Wettability versus roughness of engineering surfaces. Wear.

[15] de Castro T.C. (2017). Forensic interpretation of bloodstains on fabrics. Forensic Textile Science.

[16] Muff L.F., Luxbacher T., Burgert I., Michen B. (2018). Investigating the time-dependent zeta potential of wood surfaces. J. Colloid Interface Sci..

[17] Hasan M.B., Calvimontes A., Synytska A., Dutschk V. (2008). Effects of topographic structure on wettability of differently woven fabrics. Text. Res. J..

[18] Calvimontes A., Grundke K., Müller A., Stamm M. (2009). Advances for the topographic characterisation of SMC materials. Materials.

[19] Milašius V. (2000). An Integrated Structure Factor for Woven Fabrics Part II: The Fabric-firmness Factor. J. Text. Inst..

[20] Obendorf S.K., Klemash A. (1982). Electron Microscopical Analysis of Oily Soil Penetration into Cotton and Polyester/Cotton Fabrics. Text. Res. J..

[21] Kissa E. (1981). Mechanisms of Soil Release. Text. Res. J..

[22] Kissa E. (1981). Wetting and detergency. Pure Appl. Chem..

[23] Li S., Huang J., Chen Z., Chen G., Lai Y. (2017). A review on special wettability textiles: Theoretical models, fabrication technologies and multifunctional applications. J. Mater. Chem. A.

[24] Hubbe M.A., Chandra R.P., Dogu D., Van Velzen S.T.J. (2019). Analytical staining of Cellulosic Materials: A Review. BioResources.

[25] Chongrak K., Eric H., Noureddine A., Jean P.G. (1998). Application of Methylene Blue Adsorption to Cotton Fiber Specific Surface Area Measurement: Part I. Methodology. J. Cotton Sci..

[26] Schindelin J., Arganda-Carreras I., Frise E., Kaynig V., Longair M., Pietzsch T., Preibisch S., Rueden C., Saalfeld S., Schmid B. (2012). Fiji: An open-source platform for biological-image analysis. Nat. Methods.

